# Umbilical cord‐derived mesenchymal stem cell secretome promotes skin regeneration and rejuvenation: From mechanism to therapeutics

**DOI:** 10.1111/cpr.13586

**Published:** 2023-12-26

**Authors:** Xixian Li, Dan Zhang, Yang Yu, Liang Wang, Muxin Zhao

**Affiliations:** ^1^ Department of Plastic Surgery The Second Hospital of Dalian Medical University Dalian Liaoning China; ^2^ CAS Key Laboratory of Separation Science for Analytical Chemistry Dalian Institute of Chemical Physics, Chinese Academy of Sciences Dalian Liaoning China; ^3^ Research and Teaching Department of Comparative Medicine Dalian Medical University Dalian Liaoning China

## Abstract

How to effectively repair cutaneous wounds and promote skin rejuvenation has always been a challenging issue for clinical medicine and medical aesthetics. Current conventional medicines exhibit several drawbacks, including limited therapeutic effects, prolonged treatment periods, and high costs. As a novel cell‐free therapy, the umbilical cord‐derived mesenchymal stem cell (UCMSC) secretome may offer a promising approach for skin regeneration and rejuvenation. The UCMSC secretome is a collection of all proteins secreted by mesenchymal stem cells, including conditioned media, exosomes, and other substances. The UCMSC secretome has numerous abilities to accelerate acute wound healing, including high fibroblast and keratinocyte proliferative activity, pro‐angiogenesis, anti‐inflammation, anti‐fibrosis, and anti‐oxidative stress. Its impact on the four stages of wound healing is manifested by inducing the haemostasis phase, inhibiting the inflammation phase, promoting the proliferation phase, and regulating the remodelling phase. Furthermore, it is highly effective in the treatment of chronic wounds, alopecia, aging, and skin homeostasis disturbance. This review focuses on the clinical therapies and application prospects of the UCMSC secretome, encompassing its source, culture, separation, identification, storage, and pretreatment. Additionally, a discussion on the dosage, administration route, efficacy, and biosafety in the clinical situation is presented. This review aims to provide scientific support for the mechanistic investigation and clinical utilisation of the UCMSC secretome in wound healing and skin rejuvenation.

## Graphical abstract

This review can be separated into two sections, one on mechanisms and the other on applications. Acute and chronic wound healing, anti‐aging, hair follicle regeneration, and other skincare effects are all supported by the UCMSC secretome in a variety of ways. Different functions in four stages are skin regeneration mechanisms. The review also covers everything from secretome culture, separation, and pretreatment through dosage, administration route, efficacy, and biosafety in the clinic.

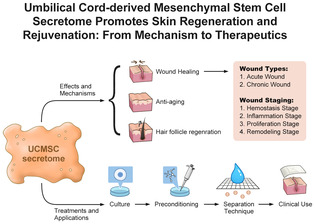

## 1 INTRODUCTION

Currently, with patients placing greater emphasis on quality healthcare, how to effectively facilitate wound recovery and improve skin aesthetics is a prevalent issue that continues to plague clinical treatment.^1^ Mesenchymal stem cells (MSCs) are applied for a cell‐based treatment method that is attractive in the field of skin regeneration, due to their helpful properties of multilineage differentiation, immunomodulation, self‐renewal, and proliferation stimulation.^2^ MSCs can be derived from a variety of sources, including bone marrow, adipose tissue, umbilical cord, amniotic membrane, and so forth.^3^, ^4^ UCMSCs have been shown in dozens of studies to be a prospective and optimal therapeutic approach in all kinds of MSCs, owing to their advantageous characteristics, including easy extraction, low cost, noninvasive collection procedures, plentiful cell content, and low immunogenicity.^2^, ^5^ UCMSCs are mainly isolated from two sections of the umbilical cord: Wharton's jelly and the umbilical vein endothelium. Due to the abundance of Wharton's jelly in the umbilical cord, it commonly used as the preferred experimental subject.^6^ Wharton's jelly MSCs have been found to contribute to skin wound healing.^7^ And even exosomes from the acellular gelatinous Wharton's jelly demonstrate effective cutaneous wound healing abilities.^8^

The primary way that UCMSCs can promote tissue regeneration is via the secretion of bioactive factors known as the UCMSC secretome or conditioned medium (UCMSC‐CM).^9^ Therefore, UCMSC‐CM has emerged as a novel and promising treatment option. Not only does human‐derived UCMSC‐CM repair skin efficiently, but other mammalian‐derived UCMSC‐CM, such as red deer‐derived UCMSC‐CM, has demonstrated similar effects in clinical trials for skin repair after laser resurfacing.^10^

Skin regeneration is the process of restoring the skin structure and basic functions, including the repair of acute wounds, burn wounds, primary wounds, chronic wounds such as diabetic wounds, and so forth. Skin rejuvenation primarily refers to the recovery of skin cosmetic functions, such as anti‐aging, hair follicle regeneration, whitening, reducing skin sensitivity, and so forth. The effects of the UCMSC secretome on skin regeneration and rejuvenation can be characterized as promoting acute wound healing mostly, promoting chronic wound healing, anti‐aging, and hair follicle regeneration.^11^, ^12^, ^13^, ^14^ The yearly expansion in demand for skin repair, as well as the rise in publications on its positive effects over the last decade, contributed to the need for this review. The study aims to elucidate the mechanisms, relevant therapeutics, and application perspectives thus far. The basic concepts, like UCMSC secretome and cutaneous wound, will then be introduced briefly.

### 1.1 UCMSC secretome

**FIGURE 1:**
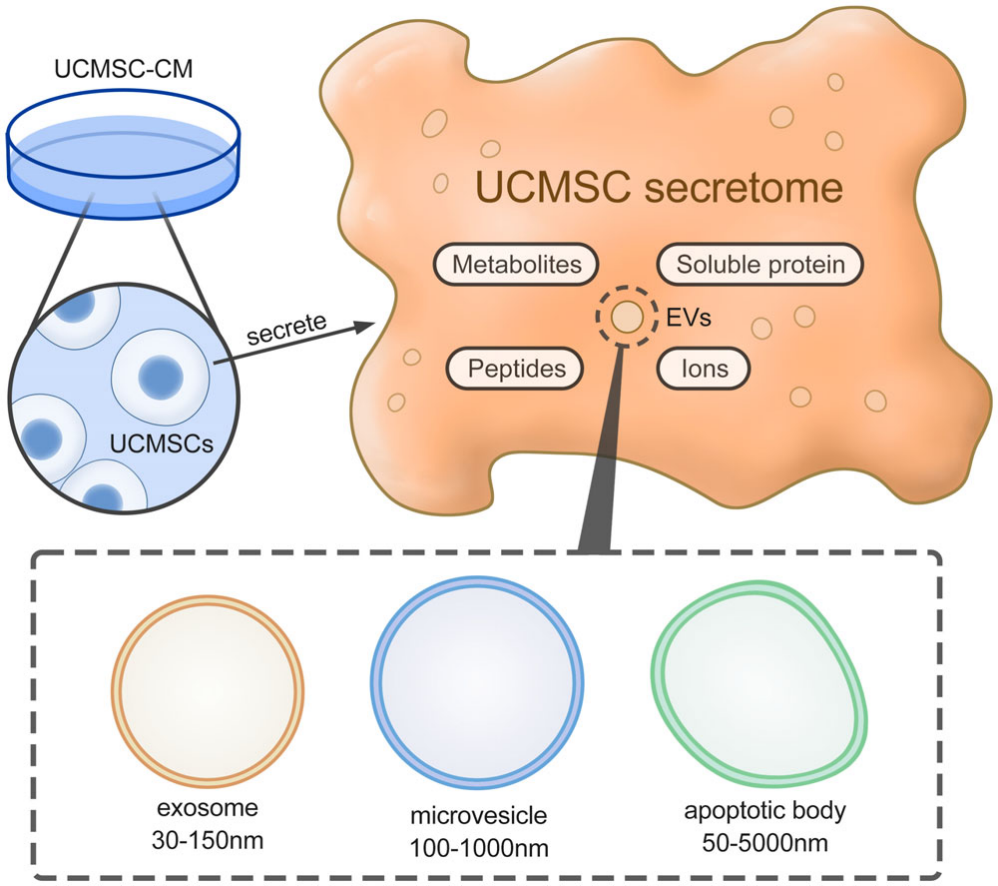
Components of the UCMSC secretome. In all figures, a square with rounded corners stands for the component. The location of squares stands for the affiliation of different components. The UCMSC secretome is the collection of all proteins secreted by UCMSCs, which is generally regarded as UCMSC‐CM. UCMSC‐EV is a kind of tiny lipid membrane vesicle in UCMSC‐CM. UCMSC‐EVs can be divided into three subtypes: exosomes, MVs, and apoptotic bodies, with different diameters, respectively, 30–150 nm, 100–1000 nm, and 50–5000 nm.

The collection of all proteins released by UCMSCs, such as growth factors, cytokines, chemokines, and so forth, is known as the UCMSC secretome. Additionally, it broadly refers to all soluble factors and extracellular vesicles, including metabolites, ions, peptides, microvesicles (MVs), and exosomes. A conditioned medium is the medium that remains after removing cells from a culture. The secretome is typically regarded as its principal part or itself, as shown in (Figure 1).^15^ Multiple physiological processes, including angiogenesis, neurogenesis, tissue repair, immunomodulation, wound healing, and anti‐fibrosis, can benefit from the MSC secretome. Compared to cell‐based therapy with stem cell transplantation, which entails the risk of infection and pro‐tumorigenicity, cell‐free therapy with secretome injection is less immunogenic, safer more trustworthy, less expensive, and more practical for clinical application.^16^, ^17^, ^18^

Umbilical cord MSC‐derived exosomes (UCMSC‐exo) are a particular kind of EV. EVs, which are tiny lipid membrane vesicles released from cells into the extracellular matrix, are involved in a variety of biological processes, including cellular communication, angiogenesis, cell migration, the development of tumour cells, and so forth.^19^, ^20^ EVs are classified into three subtypes: MVs, exosomes, and apoptotic bodies, each with its size, content, function, biogenesis, release pathways, and proteomic profiles, as shown in (Figure 1). EVs are released by almost all cell types and have been detected in plasma, saliva, milk, serum, amniotic fluid, lymphatic fluid, and other human bodily fluids. Exosomes are typically 30–150 nm in diameter, MVs are 100–1000 nm in diameter, and apoptotic bodies are 50–5000 nm in diameter. Exosomes are formed by the inward budding of the limiting membrane of early endosomes, which is followed by the formation of multi‐vesicular bodies that finally mature and are released outside the cell. MVs and apoptotic bodies are respectively generated by the outward budding and fission of the cell membrane.^21^, ^22^ Exosomes have so many useful properties that other secretomes do not have, such as better stability allowing long‐term preservation in vivo, modification ability with targeting molecules, the homing mechanism that is being internalised by target cells, and higher loading capacity of protein and RNAs.^17^, ^23^ In addition, exosomes endow with abundant DNA, miRNA, heat shock protein, angiogenesis factor, and so forth. Among them, exosome‐derived transcriptome, which includes circRNAs, lncRNAs, and miRNAs, is critical in skin repair.^24^

### 1.2 Cutaneous wound

The skin is a complicated organ with two structural layers, the dermis and the epidermis, separated by a basement membrane.^25^ A cutaneous wound is a pathological condition produced by disease, mechanical, chemical, or physiological injury to the skin. A wound can be classed as acute or chronic, depending on the cause and recovery duration of the injury. Chronic wounds recover in more than 3 months. Acute wound is often caused by physical or chemical damage, as well as surgical treatments, whereas chronic wound is typically caused by diseases such as infections, diabetes, and vascular disease.^26^, ^27^ Because the UCMSC secretome has been found to enhance cutaneous wound healing, it is essential to understand the wound healing process.^23^, ^28^

Wound healing is an intricate, dynamic, and harmonious process that involves 4 distinct and overlapping stages: haemostasis, inflammation, proliferation, and remodelling. During the haemostasis stage, damaged tissue and cells release vasoactive chemicals that promote local vasoconstriction, while platelets coagulate, activating the blood coagulation system and forming clots that stop bleeding and protect the wounded area. During the inflammation stage, mast cells produce histamine to increase neutrophil infiltration of the wounded tissue, and neutrophils begin to clear cellular debris and prevent infection. To eliminate the residual cellular debris and neutrophils, monocytes develop into macrophages. During the proliferation stage, keratinocytes move to close the wound gap. Fibroblasts move and proliferate to make granulation tissue and collagen. Subsequently, ECM components are deposited by fibroblasts and keratinocytes. New blood vessels are generated by vascular endothelial cells, and cell growth factors are released by macrophages. During the remodelling stage, vasculature degenerates. Simultaneously, fibroblasts remodel the deposited ECM and partially differentiate into myofibroblasts, which contract to smooth and reduce the wound.^29^, ^30^, ^31^

The regulation of gene and protein expression mediate the impact of the UCMSC secretome on wound healing, which is inhibitory during the inflammation stage and promotive during the proliferation stage, with the latter having the most dominating influence. During this period, epidermal stem cells, fibroblasts, and immunocytes are recruited to the wound site, where their coordinated efforts ensure smooth progression of the entire wound‐healing process.^32^ Furthermore, the UCMSC secretome stimulates the secretion of endogenous cytokine or growth factor and exerts paracrine effects.^33^

## 2 THE EFFECTS AND MECHANISM OF UCMSC SECRETOME ON WOUND HEALING

### 2.1 UCMSC secretome effects and mechanism on acute wound

#### 2.1.1 Haemostasis stage

**FIGURE 2:**
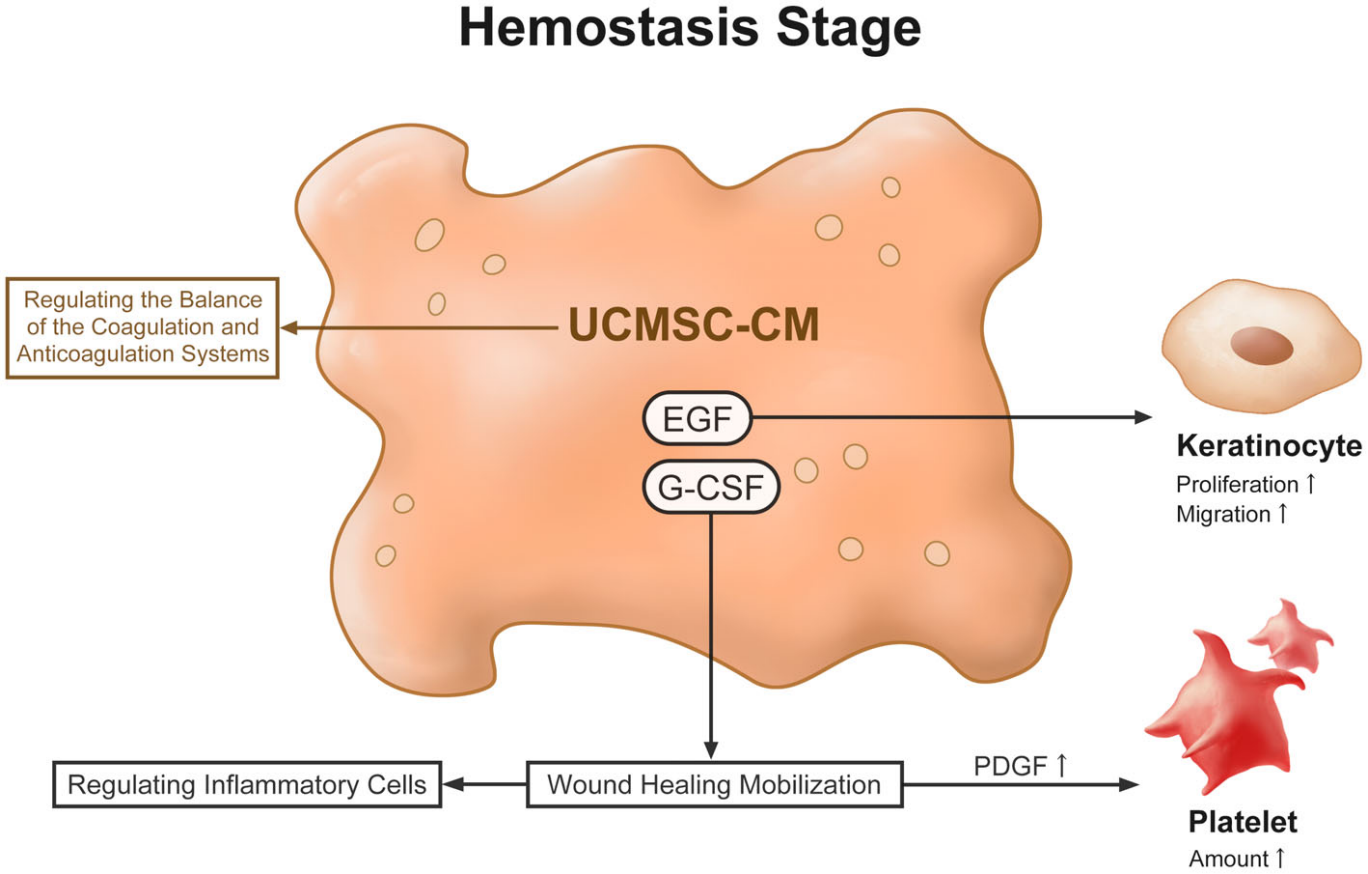
The effects and mechanism of UCMSC secretome during the haemostasis stage. In all figures, the brown colour stands for the CM effect. UCMSC‐CM during the haemostasis stage mainly regulates the balance of the coagulation and anticoagulation systems. It can lead to wound healing mobilisation to enhance the amount of PDGF and platelets.

The effect of the UCMSC secretome in the haemostasis stage has received less attention in the literature. Yet, UCMSCs have been found to regulate the balance of the coagulation and anticoagulation systems as well as cure immune‐related thrombophilia.^34^ Miranda et al. also discovered that the EGF family in UCMSC‐CM increases autocrine secretion of itself and enhances the promitogenic and motogenic‐inducing capacities of keratinocytes at this stage. G‐CSF in UCMSC‐CM is in charge of encouraging the mobilisation of cells linked to tissue regeneration, such as the proplatelet effect brought on via the secretion of PDGF and regulating inflammatory cells like macrophages, neutrophils, and mast cells (Figure 2).^35^

#### 2.1.2 Inflammation stage

**FIGURE 3:**
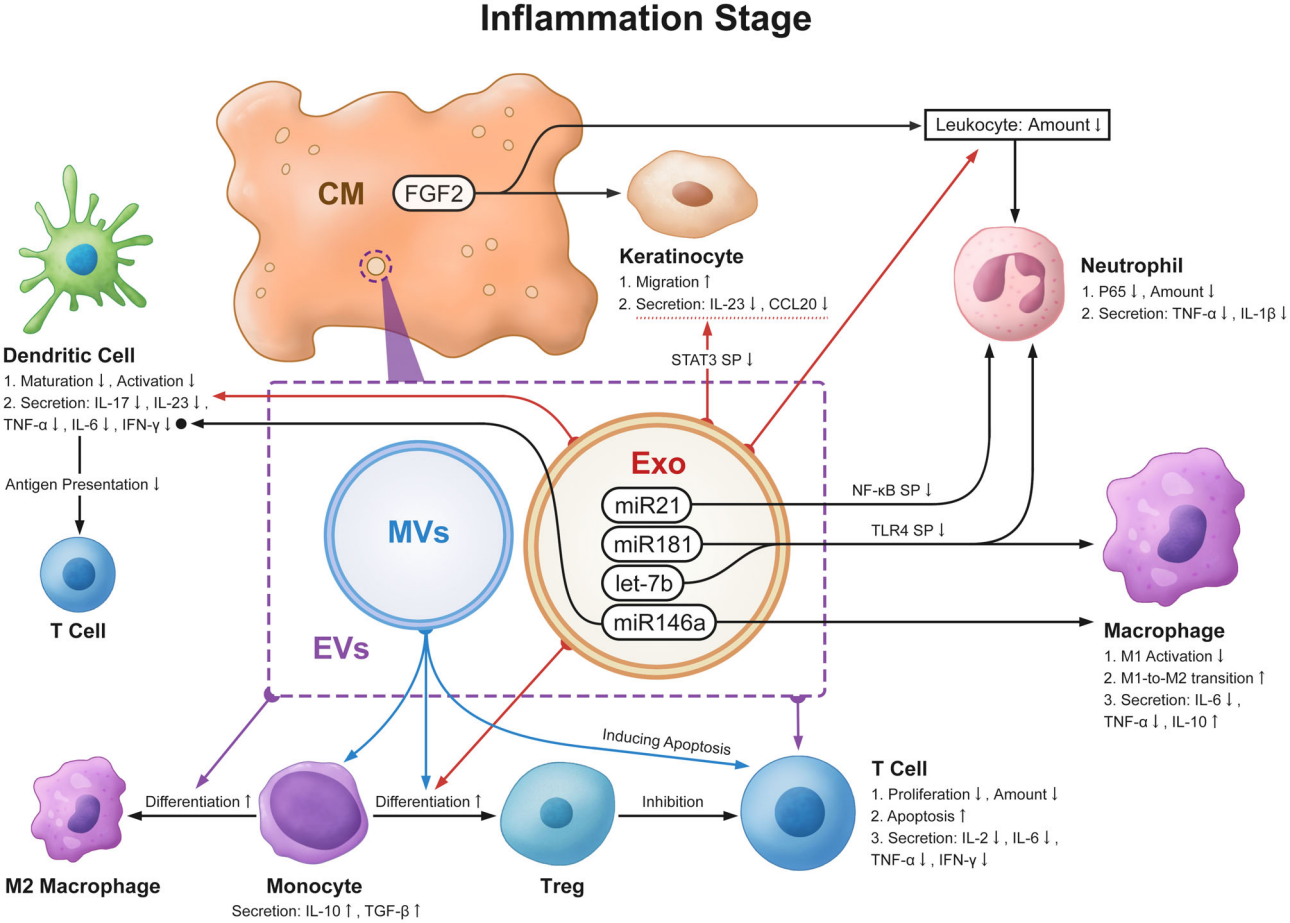
The effects and mechanism of UCMSC secretome during the inflammation stage. (Abbr. SP, signalling pathway) In all figures, the red, blue, and purple colours, respectively, stand for exosomes, EVs, and MVs effects. In all figures, black spots and dashed underlines stand for the fact that the substance only affects or is affected by the guided texts, not the whole cell. During the inflammation stage, the UCMSC secretome can affect inflammatory cell secretion, which causes a reduction of inflammatory factors, an enhancement of anti‐inflammatory factors, and a weakening of antigen presentation. It can also decrease T cell and leukocyte numbers and proliferation and regulate macrophage, monocyte, and dendritic cell activation and differentiation. Besides, the migration of keratinocytes is promoted.

During this stage, the UCMSC secretome mainly inhibits the excessive inflammation of wound repair. Thus, if it is used in the treatment, precautions need to be taken to both actively prevent infection and safeguard the surface of a wound. UCMSC‐exo has potent immunomodulatory effects in that it can control the activation of immune cells and suppress the expression of inflammatory cytokines. Their mechanisms are summarised in (Figure 3). ICAM‐1 is central to the regulation of the inflammatory process. UCMSC‐exo can help to control inflammation by upregulating the ICAM‐1 gene expression.^33^ MiRNAs, like miR‐21, miR‐146a, and miR‐181, enriched in UCMSC‐exo, play a major anti‐inflammatory role. IL‐6 and TLR signalling pathways are potential downstream targets for their activity.^36^, ^37^, ^38^

##### Leukocyte

The primary function of leukocytes is to combat pathogens. Neutrophils constitute the majority of leukocytes. Their persistent existence causes chronic inflammation and chronic refractory wounds, including diabetic foot ulcers, pressure sores, and leg vein ulcers.^31^ UCMSC‐exo decreases the total amount of leukocytes and reduces protein expression of P65 and p‐P65. It also downregulates the TLR4 and NF‐κB signalling pathways, thereby inhibiting the release of TNF‐α and IL‐1β.^36^ Among them, exosomal miR‐21 was shown to silence PTEN and GSK3β genes, thus inhibiting the function of NF‐κB activation.^37^

##### T cell

T cells check the skin for infections, release cytokines, and deliver epidermal cell signals, at this stage. In contrast, regulatory T cells (Treg) possess active regulatory suppression of T cells to function as anti‐inflammatory.^31^, ^39^ UCMSC‐EVs were discovered by Marta et al. to have the ability to restrain body immunity. The growth of T cells and the release of cytokines like IL‐2, IL‐6, TNF‐α, and IFN‐γ were significantly inhibited by UCMSC‐EVs.^40^ In addition, the suppressive impact of UCMSC‐EVs was more prominent compared to adipose or bone marrow‐derived MSC‐EVs.^41^ Mokarizadeh et al. found that MSC‐MVs can inhibit auto‐reactive lymphocyte proliferation and stimulate monocytes to release IL‐10 and TGF‐β to induce Treg formation.^42^ UCMSC‐exo can promote monocyte‐to‐Treg differentiation.^43^ If the exosome is produced by TGF‐β and IFN‐γ stimulation, the process will function more efficiently. The differentiation may be associated with an increase in several cytokines, such as IDO and IL‐10.^44^

##### Macrophage

Macrophages are primarily M1 phenotype at this stage. They phagocytose cellular debris and residual neutrophils, and release pro‐inflammatory cytokines such as IL‐1β, IL‐6, and TNF‐α, to avoid infection.^31^ LPS‐preconditioned UCMSCs enhance the paracrine protective effects and regenerative properties. LPS‐preconditioned UCMSC‐derived exosomes also have a superior ability to regulate inflammation and regeneration, which is linked to a coordinated effect on macrophages. Because of the increased let‐7b content in exosomes affected by LPS, exosomal let‐7b negatively regulates the downstream TLR4 signalling pathway to transition macrophages to the M2 state, balancing the polarisation of macrophages to resolve chronic inflammation.^45^ Exosomal miR‐181c can attenuate burn‐induced hyperinflammatory reactions in macrophages by downregulating the TLR4 signalling pathway while increasing the expression of IL‐10. IL‐10 is considered an anti‐inflammatory cytokine.^36^ Exosomal miR‐146a can also polarise macrophages to the M2 phenotype, enhancing immunomodulatory functions.^46^ In addition, UCMSC‐EVs can encourage the differentiation of monocytes into macrophages with the M2 phenotype.^40^

##### Dendritic cell

Dendritic cells can be activated to release IFN‐α and IFN‐β to induce acute inflammation. They are primarily involved in antigen presentation that triggers T‐cell responses.^31^ UCMSC‐exo can decrease antigen presentation from dendritic cells to T cells via downregulating cellular IL‐17 and IL‐23 secretion. It also prevents dendritic cell maturation and activation to lessen cutaneous inflammation.^47^ Exosomal miR‐146a, taken up by dendritic cells, limits endotoxin‐induced inflammation in mice and suppresses the expression of inflammation‐related genes like TNF‐α, IL‐6, and IFN‐γ.^38^

##### Keratinocyte

At this stage, keratinocytes can secrete pro‐inflammatory cytokines and chemokines to be involved in the inflammatory response. For instance, the release of CCL20 can recruit Th17 cells to the site of the injury. Via downregulating the STAT3 signalling pathway, UCMSC‐exo can inhibit the secretion of IL‐23 and CCL20 from Keratinocytes thereby suppressing the inflammatory response.^47^ Through a chemotactic process, the FGF‐2 present in UCMSC‐CM is crucial for the morphogenesis and organisation of suprabasal keratinocytes and also causes leukocyte recruitment on the endothelium (Figure 3).^35^

#### 2.1.3 Proliferation stage

**FIGURE 4:**
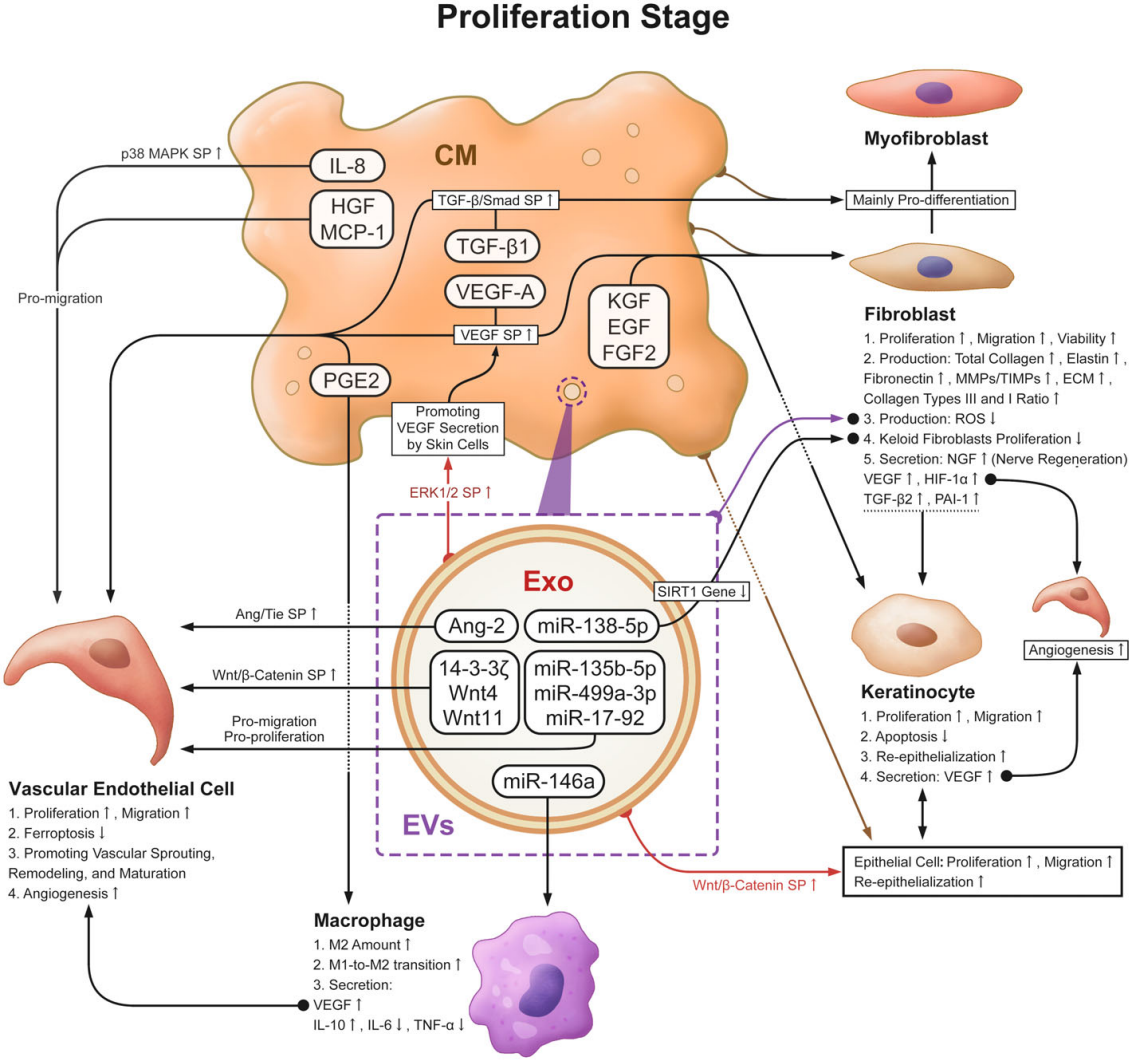
The effects and mechanism of UCMSC secretome during the proliferation stage. ①Vascular endothelial cell: Angiogenesis is the greatest effect of the UCMSC secretome at this stage. Not only can it promote vascular endothelial cell migration and proliferation, but other skin cells are also activated to secrete VEGF and promote angiogenesis. ②Fibroblast and keratinocyte: Their migration and proliferation are enhanced to promote collagen synthesis, ECM deposition, and re‐epithelialization. ③Macrophage: The phenotype of macrophages changes from M1 to M2. The UCMSC secretome can reduce inflammation‐related gene and protein expression.

The majority of pertinent papers discussed the promotion during the proliferation stage, including angiogenesis, re‐epithelialization, and so forth. The most overt proliferative effects were seen in fibroblasts, epithelial cells, and keratinocytes.^48^ Likewise, since activated fibroblasts are largely responsible for the formation of granulation tissue, the UCMSC secretome is also strongly favourable to forming granulation tissue Nevertheless, pro‐angiogenesis is the predominant function in the proliferation stage, and their mechanisms are summarised in (Figure 4).

##### Vascular endothelial cell

The UCMSC secretome possesses pro‐angiogenic capacity via modulating the proliferation and migration of vascular endothelial cells to promote vascular sprouting, remodelling, and maturation in the wound, thereby increasing the number of capillaries and microvessels.^49^ VEGF plays an essential role in angiogenesis and engages in the VEGF pathway. However, the secretome acted differently in various experiments via the same pathway. VEGF‐A can be secreted directly and independently into adjacent cells through the UCMSC paracrine mechanisms to foster endothelial cell migration. Studies have demonstrated the UCMSC secretome contains little to no VEGF‐A compared to other sources like bone marrow and adipose tissue.^50^, ^51^, ^52^ On the contrary, Sandra S. Edwards et al. suggested that UCMSCs are capable of releasing VEGF‐A into CM, where it subsequently functions as a promoter.^53^ While Chinnici et al. hypothesised that their EV‐derived miRNAs enhance the endogenous VEGF‐A.^54^ VEGF‐A has been revealed to be expressed in a specific temporal pattern in wounds. Capillary development and differentiation are at their peak 3–7 days after wounding. The pro‐angiogenic expression then steadily declines until it is almost absent during the remodelling stage.^35^ Moreover, VEGF‐A is unique in that it affects wound healing in a multiple, compound, cascade reaction pattern, such as vascularity, epithelialization, and collagen deposition.^33^

In UCMSC‐CM, TGF‐β1 assists the angiogenic growth via the TGF‐β/Smad signalling pathway and is required for fibroblast differentiation into myofibroblasts.^35^, ^55^ IL‐8, HGF, and MCP‐1 in UCMSC‐CM play a part in driving vascular endothelial cell migration. IL‐8 can induce cytoskeletal rearrangement and directional migration of endothelial cells by activating the р38MAPK signalling pathway.^56^ The concentration of HGF and MCP‐1 can affect the migratory capacity of endothelial cells.^50^, ^57^

The pro‐angiogenic mechanism of UCMSC‐exo has received the most attention. In the study by Yiyao Zhang et al., UCMSC‐exo can control the ERK1/2 pathway to elevate the levels of VEGF secreted by fibroblasts, keratinocytes, and inflammatory cells for angiogenesis.^58^ Currently recognised pro‐angiogenic components found in exosomes include Ang‐2,^59^ Wnt‐4,^49^ Wnt‐11,^60^ 14‐3‐3ζ,^61^ PGE2^62^ and various MiRNAs, of which miR‐146a,^46^ miR‐17‐92,^63^ miR‐135b‐5p and miR‐499a‐3p^64^ are proven. The most characteristic growth factor, Ang‐2, is a member of the Ang/Tie signalling pathway, one of the key pro‐angiogenic pathways. Ang‐2 can be enriched in exosomes and delivered into endothelial cells to enhance angiogenesis. In vitro, an experiment has shown that overexpression of Ang‐2 in exosomes has therapeutic and pro‐angiogenic benefits on cutaneous wound healing, whereas knockdown of the Ang‐2 gene produces the opposite effect.^59^ Exosomal MiR‐17‐92 can enhance endothelial cell proliferative and migratory activity and resist cell ferroptosis.^63^ MiR‐135b‐5p and miR‐499a‐3p, which are highly expressed in blue light‐treated exosomes, induce capillary formation both in vitro and in vivo. They together inhibit the expression of the MEF2C gene to promote the proliferative and migratory activity of endothelial cells.^64^

Additionally, the Wnt/β‐Catenin signalling pathway is critical for angiogenesis. Because the lipid modification of Wnt prevents it from spreading over a distance among cells, exosome‐like particles need to be the main carriers of Wnt molecules if Wnt/β‐Catenin is to have a long‐range effect. The exosomes and EVs can carry Wnt on their surface, thus inducing Wnt signalling activity in target cells. Wnt4, Wnt11, and 14‐3‐3ζ in UCMSC‐exo can trigger the Wnt/β‐Catenin signalling pathway to support angiogenesis and wound healing. CD29 and α4 integrin may be involved in the Wnt4 activation of this signalling pathway.^49^, ^60^, ^61^

##### Fibroblast

The effects of the UCMSC secretome on fibroblasts are mainly to promote their migration, proliferation, elastin synthesis, and the entire production of granulation tissue.^65^, ^66^ With the UCMSC‐CM treated, there is a substantial increase in the number of dermal fibroblasts, cell viability, total collagen, elastin, and fibronectin levels.^33^ UCMSC‐CM can activate the expression of genes such as TGF‐β2, HIF‐1α, and PAI‐1 that are involved in re‐epithelialization, neovascularization, and fibre regeneration.^7^ As reported by Meirong Li et al., after receiving UCMSC‐CM therapy, the fibroblast‐to‐myofibroblast differentiation was reduced and the collagen types III and I ratio and the MMPs/TIMPs ratio of adult fibroblasts were increased. These characteristics imply that the enhancement of wound repair may result from the transformation of adult fibroblasts into fetal fibroblasts.^65^

UCMSC‐CM has many beneficial components that enhance fibroblasts, such as FGF2, EGF, KGF, and VEGF‐A.^35^, ^67^, ^68^ VEGF‐A in UCMSC‐CM can target to stimulate ECM synthesis, granulation tissue formation, fibroblast proliferation, and migration, deep in the wound.^69^ FGF2, EGF, and KGF have been shown to promote fibroblasts and keratinocytes proliferative activity, re‐epithelialization, and ECM formation and remodelling.^35^, ^67^, ^68^ However, dermal fibroblast responses to UCMSC‐EVs and UCMSC‐CM vary. A study by Duc Minh Vu et al. illustrated that UCMSC‐EVs cannot significantly promote proliferative and migratory activity, but only TGFβ‐stimulated UCMSC‐EVs exhibit the activity and increase ECM proteins synthesis to fight against aging.^70^ In addition, UCMSC‐exo can recruit fibroblasts and stimulate them to produce NGF, thus promoting cutaneous nerve regeneration.^20^

In chronic wounds, prolonged local high glucose microenvironment activates p21 and p16 expression in a ROS‐dependent manner to cause fibroblast senescence. It also slows fibroblast growth, migration, and differentiation via downregulating the TGF‐β/Smad signalling pathway. UCMSC‐CM can decrease the ROS overproduction from fibroblasts, which promotes the function and antagonised the dysfunction of their differentiation to myofibroblasts.^71^ UCMSC‐EVs have also been reported to have antioxidant activity which lowers oxidative stress in fibroblasts and heals wounds like diabetic ulcers that are damaged by cellular oxidative stress.^72^

Finally, the UCMSC secretome can inhibit keloid. The growth of keloids is linked to the transformation of normal fibroblast into keloid fibroblast and the tumour‐like proliferative activity. UCMSC‐CM can effectively inhibit the proliferation of keloid fibroblasts.^73^, ^74^ Besides, KGF in UCMSC‐CM plays a positive effect on reducing scar formation and may be responsible for its scar inhibitory function.^35^, ^67^ MiR‐138‐5p, contained in UCMSC‐exo, plays a key role in reducing pathological scarring, and it can inhibit scar development by downregulating the SIRT1 gene to reduce the growth of scar fibroblasts.^15^

##### Epithelial cell

The UCMSC secretome shows a strong pro‐epithelialization at this stage. In murine experiments, it made faster and thicker epidermal growth in wounds compared to those without it.^75^, ^76^, ^77^ It has been demonstrated that PDGF, FGF‐2, and TGF‐β enhance the proliferation and migration of epithelial cells to form an epidermal layer and seal the wound.^78^ The re‐epithelialization effect via the UCMSC secretome may be related to these contained cytokines. According to an in‐vivo experiment, the role of UCMSC‐exo in stimulating Wnt/β‐catenin signalling pathway activation is crucial for the proliferation and re‐epithelialization of epithelial cells in wounds.^79^

Keratinocytes, the predominant component of the skin epithelium, have significance for re‐epithelialization. The UCMSC secretome mainly promotes the proliferation and migration of keratinocytes.^28^, ^47^, ^66^ Keratinocytes will form cell–cell straps when they migrate to the wound surface.^80^ The cell–cell strap is a collagen strap formed by the cell traction force between keratinocytes. For assistance in cell migration, the cells pull the collagen fibres into a sort of bundle around themselves, which UCMSC‐CM can facilitate.^28^, ^81^ Furthermore, UCMSC‐CM has an even greater capacity to promote keratinocyte migration than fibroblasts. It contains EGF, FGF‐2, and KGF, which are responsible for the early induction of keratinocyte migration and function.^35^ KGF not only boosts keratinocyte proliferation but also has a footprint on the early function of keratinocytes, which results in the regulation of VEGF gene expression and plays an integral part in the pro‐angiogenic effect during the proliferation and remodelling stage.^82^ UCMSC‐exo can sharply boost keratinocyte proliferation and migration in a time‐ and dose‐dependent manner. In addition, it also prevents H_2_O_2_‐induced apoptosis in keratinocytes via raising the levels of PARP‐1 and ADP‐ribose, which inhibits AIF‐induced nuclear translocation.^77^

##### Macrophage

As the anti‐inflammatory response subsides, the prevailing phenotype of macrophages in the proliferation stage changes from M1 to M2, and their early function changes to vasculature development. Microvascular density is positively correlated with an increase in the number of macrophages at this stage. Additionally, macrophages can induce a transition from fibroblasts to myofibroblasts in the middle to late stages.^31^ PGE2 derived from UCMSC‐CM and UCMSC‐exo can drive M1 macrophages to develop an anti‐inflammatory M2 phenotype, increasing the amount of M2 macrophages. They may modify the expression of M2 macrophage‐derived cytokines, such as increased IL‐10 and VEGF production and decreased IL‐6 and TNF‐α secretion. These alterations in cytokine release can improve the local microenvironment of vascular endothelial cells, enhance their function, and hence accelerate angiogenesis and collagen deposition which aids wound healing.^62^, ^83^ MiR‐146a, a MiRNA in UCMSC‐exo that can help phenotypic switch of M1 to M2 macrophages, can thereby promote angiogenesis (Figure 4).^46^

#### 2.1.4 Remodelling stage

**FIGURE 5:**
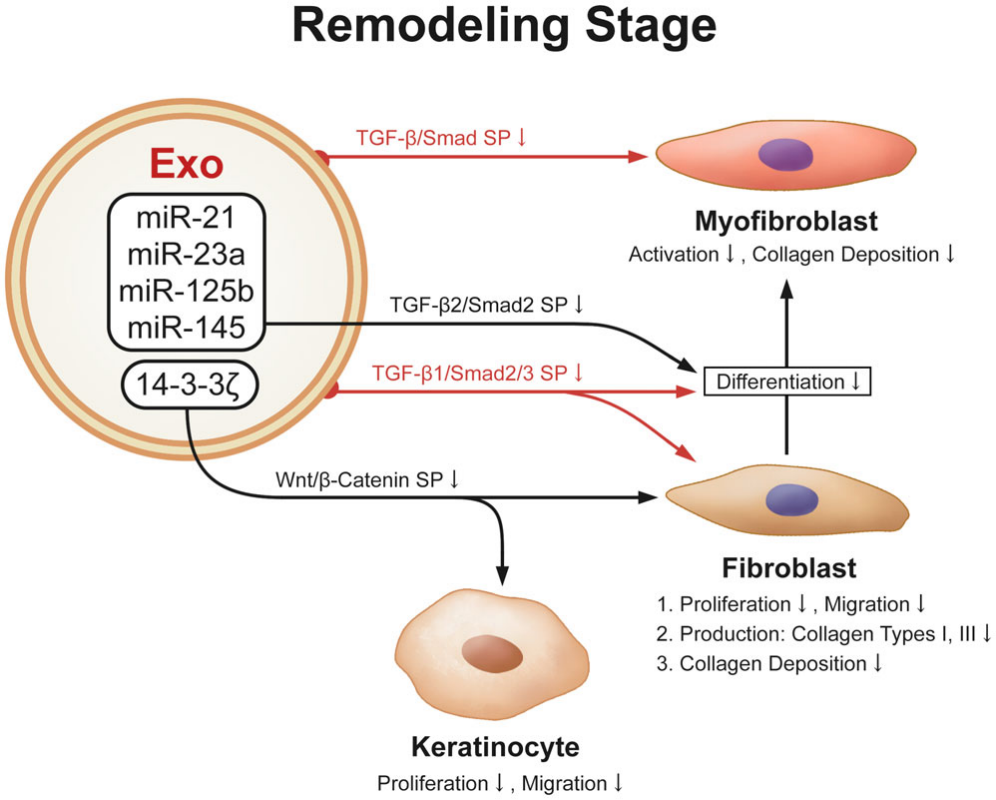
The effects and mechanism of UCMSC secretome during the remodelling stage. UCMSC‐exo can lessen the differentiation from fibroblast to myofibroblast and the cytoactive of fibroblasts and myofibroblasts, during the remodelling stage, mainly via the TGF‐β/Smad signalling pathway.

The fundamental role of the UCMSC secretome during the remodelling stage is evident in the inhibition of the differentiation process of fibroblasts to myofibroblasts and the ability to restrict scar formation.^65^, ^84^, ^85^ Although myofibroblasts can shrink the wound and give the skin greater tension so that it can resume its previous function, excessive accumulation can lead to further local contracture and fibrosis, resulting in scar formation.^30^ UCMSC‐exo can inhibit the production of type I and type III collagen by fibroblasts. Through blocking the TGF‐β1/Smad2/3 signalling pathway, fibroblast transition to myofibroblasts is prevented.^84^ Fang et al. took a step further, discovering that exosomal miRNAs like miR‐21, miR‐23a, miR‐125b, and miR‐145 play a vital role in inhibiting this transition via suppressing the TGF‐β2/Smad2 signalling pathway, as shown in (Figure 5).^85^ UCMSC‐exo may additionally alleviate this transition via downregulating the TGF‐β/Smad signalling pathway to attenuate myofibroblast activation and collagen deposition, hence reducing dermal fibrosis. As a result, the antifibrotic effect of the UCMSC secretome at this stage could be used to treat autoimmune dermal fibrotic diseases.^86^

The Wnt/β‐Catenin signalling pathway is also crucial during the remodelling stage (Figure 5). The exosomal protein 14‐3‐3ζ can mediate YAP and p‐LATS binding by generating a complex that promotes YAP phosphorylation, thereby coordinating this signalling pathway to limit keratinocytes and fibroblasts over‐expansion and collagen deposition in skin regeneration. Therefore, UCMSC‐exo not only acts as an ‘accelerator’ of the Wnt/β‐catenin signal to repair damaged skin tissues during the proliferation stage but also coordinates the management of skin regeneration during the remodelling stage via regulating YAP as a ‘brake’ of this signal.^61^

### 2.2 UCMSC secretome effects and mechanism on chronic wound

Chronic wounds are those that are older than about 3 months and fail to restore the anatomical and functional integrity of the wounded location through a prompt and orderly healing process. Chronic wounds can be classified into four categories: pressure ulcers, diabetic ulcers, venous ulcers, and arterial ulcers.^87^ Contrary to acute wounds, such as burns and surgical wounds, which can recover on their own, individuals with chronic wounds, like diabetic trauma, require an extremely extended period to heal. Therefore, it differs considerably from an acute wound in terms of the mechanism of wound development. Chronic wounds have lower numbers of skin stem cells than acute wounds and higher quantities of proinflammatory cytokines, ECM proteases, ROS, and senescent cells.^88^, ^89^ This dysregulation keeps the wound from progressing to the proliferation stage and keeps it stuck in the inflammation stage.^90^ The UCMSC secretome has been reported most frequently concerning the management of arterial and diabetic ulcers.

The main causes of diabetic wounds are multiple drug‐resistant bacterial infections, vasculopathy, hypoxia, and oxidative stress damage to the microenvironment.^30^, ^91^ The mechanism of the UCMSC secretome in the treatment of diabetic ulcers is also related to the amelioration of these reasons. For instance, as was already indicated, UCMSC‐CM and UCMSC‐EVs can lower fibroblast ROS generation and decrease oxidative stress damage from hypoxia. According to the research by Fong et al., UCMSC‐CM was more effective at treating wounds than UCMSC transplantation at promoting the repair of diabetic ulcers.^92^ In a more thorough study, Fong et al. found that UCMSC‐CM treatment of diabetic wounds can improve re‐epithelialization and vascular supply, manifested as the increased amount of cell density, sebaceous glands, and hair follicles, as well as the positive expression of ICAM‐1, TIMP‐1, VEGF‐A, and keratinocyte markers (cytokeratin, involucrin, and filaggrin) in the wound bed.^33^ In addition, UCMSC‐CM activates macrophage conversion to M2 and significantly enhances diabetic vascular endothelial cell function, including production, migration, and chemotaxis.^62^ UCMSC‐exo acts somewhat similarly to CM in that it accelerates diabetic wound healing by modulating the ability of vascular endothelial cells to respond to oxidative stress damage and enhancing angiogenesis.^93^ Many experiments confirmed that circHIPK3 is increased in type 2 diabetes mellitus. Overexpressing UCMSCs‐derived exosomal circHIPK3 can directly bind miR‐20b‐5p, promoting the synthesis of Nrf2 and VEGF‐A. It enhances revascularization and wound healing, revealing an attractive therapeutic function in diabetic foot ulcers.^94^

Lower extremity ulcers, primarily arterial, are brought on by lower extremity ischemia. Zhang et al. discovered that miR‐24 is a key miRNA component in UCMSC‐exo that knockdowns B2M. MiR‐24 is loaded into UCMSC‐exo and can be intramuscularly injected into the site of injury to improve blood perfusion, muscle, and motor function to prevent the development of arterial ulcers.^95^

## 3 THE EFFECTS AND MECHANISM OF UCMSC SECRETOME ON SKIN REJUVENATION

### 3.1 UCMSC secretome effects and mechanism on skin aging

Skin aging is most visually manifested as cumulative changes in skin structure, function, and appearance, such as increased wrinkles, sagging, decreased elasticity, capillary dilation, and abnormal skin pigmentation. In contrast to other body organs, the skin is not only affected by the natural aging process but is also more influenced by various environmental factors, particularly UV radiation.^96^ The anti‐aging effect of the UCMSC secretome is mainly to reduce UV damage and weaken photoaging.

According to research on keratinocytes, UCMSC‐CM can combat photoaging by lowering apoptosis, reducing the generation of ROS, and enhancing cell motility. In this effect, MYC, IL‐8, FGF‐1, and EREG are the key genes engaged in anti‐photoaging, and C‐FOS, C‐JUN, TGF‐β, p53, FGF‐1, and cell cycle protein A2 are the major proteins involved in anti‐photoaging.^97^ A study by Liu et al. found that UCMSC serum‐free conditioned medium can prevent UVA‐ and UVB‐induced photoaging. The principle is that it can minimise UVA‐induced cell mortality by inhibiting UV‐induced suppression of SOD and GSH‐Px activities, with blocking the upregulation of malondialdehyde.^98^

Furthermore, UCMSC‐EVs dramatically increase dermal fibroblast proliferation, shield cells from UVB‐induced cell death and cell‐cycle arrest, decrease the number of senescent cells, and significantly limit UVB‐induced ROS formation.^72^ Similar antioxidant and anti‐inflammatory effects were produced by UCMSC‐exo on UV‐induced DNA damage and apoptosis. UCMSC‐exo contains 14‐3‐3ζ protein as one of the active components. It can encourage autophagy activation and exert cytoprotective functions by regulating the SIRT1‐dependent antioxidant pathway, thus reducing UV and H_2_O_2_ damage to the skin.^99^

### 3.2 UCMSC secretome effects and mechanism on hair regeneration

Hair loss is caused by many factors, including hormonal changes, nutritional deficiencies, genetics, medications, inflammation, injury, and even surgery.^21^ The UCMSC secretome may hold the key to finding a non‐drug, non‐steroidal, and non‐aggressive solution to the problem of treating hair loss. Studies in animals and clinical trials supported the efficacy of UCMSC‐CM for hair regrowth, potentially as a result of its abundance of growth factors, cytokines, and beneficial proteins. Positive treatment of hair loss was observed in 86.6% of the subjects.^100^ The study by Dong et al. suggests that the effective component in UCMSC‐CM may be Wnt7a, which acts synergistically with UCMSC to promote hair follicle regeneration. The Wnt protein family is also thought to play a role in the development of skin appendages like hairs.^101^

### 3.3 UCMSC secretome effects and mechanism on other skin rejuvenation functions

The UCMSC‐CM and UCMSC‐exo also both perform a variety of other tasks to enhance skin health. The study by Park et al. confirmed that UCMSC‐CM has melanin‐reducing, anti‐cellular oxidative stress and anti‐wrinkle effects. They also identified 18 cytokines associated with skin condition improvement in UCMSC‐CM, including amphiregulin, bFGF, EGF, GDNF, HGF, IGFBP‐4, IGFBP‐6, IGF‐1, and M‐CSF, all of which can help skin lightening and anti‐aging.^102^ A study by Xin Wang et al. also found that UCMSC‐CM can enhance the skin barrier and effectively regulate skin cell apoptosis, detoxification, and other physiological functions. It is beneficial for restoring skin homeostasis and has the potential to treat atopic dermatitis and acne.^103^ Finally, UCMSC‐exo can reduce skin sensitivity and restore skin barrier function. In clinical use, it ameliorates roughness, erythema, tension, burning, and itching in patients with skin sensitivity, and treats melasma.^104^, ^105^

## 4 CLINICAL THERAPEUTICS AND APPLICATION PROSPECTS OF UCMSC SECRETOME ON SKIN REGENERATION AND REJUVENATION

### 4.1 Modification and preparation of UCMSC secretome

The UCMSC secretome has demonstrated recovery capacity in animal models, such as deep second‐degree burn, third‐degree burn, and full‐thickness skin injury mice models. It is also beginning to see substantial use in clinical treatment. Modification of the UCMSC secretome can improve its therapeutic potential, as shown in (Table 1). We will summarise an outlook on its prospective employment, especially the modification and preparation of UCMSC secretome from the studies in vivo.

**TABLE 1:** Improvement methods of the UCMSC secretome.

References	Secretome type	Improvement method	Improvement content	Positive effect
[107, 108]	Exosome	Culture mode	3D	Wound healing and angiogenesis
[107]	Exosome	Culture mode	Serum‐free	Reducing UV damage
[109, 110]	CM, Exosome	Culture mode	Hypoxia	Diabetic wound healing
[19]	EV	Separation technique	Proper TFF	Reducing oxidative stress damage
[60]	CM	Drug stimulation	DIM	Wound healing
[70]	EV	Drug stimulation	TGFβ	Fibroblast proliferative and migratory activity
[115, 116]	CM	Drug stimulation	IFN‐γ, TNF‐α	Wound healing
[117]	CM	Gene modification	Antimicrobial peptide	Antibacterial capability
[118]	CM	Gene modification	JAM‐A	Diabetic wound healing
[119]	Exosome	Load	Iron oxide nanoparticle	Recovery efficiency
[120]	Exosome	Load	Endothelial NO synthase	Wound healing and remodelling the immune microenvironment
[58]	Exosome	Scaffold	Nano‐hydrogel	Wound healing and angiogenesis
[125]	Exosome	Scaffold	PF‐127 hydrogel	Chronic diabetic wound healing
[126]	Exosome	Scaffold	Gelatin methacryloyl hydrogel	Fractional laser injury wound healing
[127]	Exosome	Scaffold	Silk fibroin and sericin composite hydrogel	Wound healing
[128]	CM	Scaffold	HA gel	Treating diabetic foot ulcer
[129]	Exosome	Scaffold	Methacrylated HA	Wound healing and angiogenesis
[130]	Exosome	Combined therapy	Aloe‐emodin	Treating leishmaniasis
[131]	Exosome	Combined therapy	Sponge spicules	Treating photoaging
[122]	Exosome	Combined therapy	Microneedle, fractional laser, or radiofrequency	Treating melasma

#### 4.1.1 Culture condition

The culture condition of UCMSCs has been optimised in some experiments. The exosomes derived from 3D culture or serum‐free culture UCMSCs have a better ability to promote wound healing and angiogenesis.^106^, ^107^ A study by Liu et al. note the 3D culture UCMSC‐derived exosomes are effective in decreasing the UVB‐induced damage and MMPs expression in photoaged keratinocytes.^108^ Moreover, hypoxia can enhance the exosome‐mediated paracrine function of MSCs. The UCMSC cultured under hypoxic conditions, as well as their CM and exosomes, have specific and improved regenerative capabilities on diabetic wounds.^109^, ^110^ Hendrawan et al. discovered that hypoxia‐induced UCMSC‐CM had a larger favourable influence on the re‐epithelialization and collagen formation processes in wound healing than antibiotic treatment.^110^ In addition, hypoxic microenvironment culture can mimic the trauma environment. Zhang et al. demonstrated that hypoxia‐induced UCMSC‐exo‐derived miR‐125b inhibited the expression of tumour protein p53 inducible nuclear protein 1, thereby lessening wound hypoxia‐induced cutaneous cell apoptosis.^109^

#### 4.1.2 Separation technique and storage

There are two main methods for separating umbilical cord stem cell‐derived exosomes from the conditioned medium: ultracentrifugation (UC) and tangential flow filtration (TFF). Some experiments use a combination of these two methods to improve purity.^75^ UC is not suitable for higher purity extraction.^23^ And the separation yield of utilising proper TFF is two orders of magnitude higher than that of using UC.^111^ It has been reported that TFF‐extracted UCMSC‐EVs have a higher recovery rate of keratinocytes with oxidative stress damage than those extracted using other methods.^19^ For higher production, the decomposition of UCMSCs by ultrasonication can produce UCMSC‐EVs, which exhibit the same skin rejuvenating properties.^112^ The primary approach for identifying UCMSCs and their exosomes is flow cytometry. Exosomes can be recognised by observing their shape and size under an electron microscope, and by examining their surface markers via enzyme‐linked immunosorbent assay or western blotting.^60^, ^113^ UCMSC‐EVs can be stored in lyophilization at −20°C and −80°C for up to 4–6 weeks, maintaining their anti‐inflammatory and vascular activities, miRNAs, and lncRNAs.^114^

#### 4.1.3 Preconditioning

Pretreatment of the secretome can improve the efficiency of therapies such as drug stimulation, gene modification, and being loaded by active ingredients. Numerous experiments make use of drug stimulation of the secretome. For instance, DIM‐treated UCMSCs can increase their CM recovery capacity in wound healing. DIM can enhance the stemness of UCMSCs themselves through autocrine signalling of exosome‐derived Wnt11.^60^ TGFβ‐stimulated UCMSC‐EVs can exhibit fibroblast proliferative and migratory activity that is not present without stimulation.^70^ If UCMSCs are exposed to proinflammatory cytokines such as IFN‐γ and TNF‐α, their CM can be beneficial for wound healing via enhancing macrophages migration and M2 polarisation.^115^ Furthermore, a study by Lihui Tai et al. found that TNF‐α‐treated UCMSC secretome, which included higher levels of inflammation‐related FGFb, VEGF, PDGF, and IL‐6 compared to untreated group, triggered an increase in MMP13 secretion in keratinocytes.^116^

In addition, genetic modifications can bring new functionality. UCMSCs can generate antimicrobial peptides after being transfected with the hCAP‐18/LL‐37 gene. As a result, their CM has antibacterial capability and promotes wound repair.^117^ UCMSCs were transfected with lentivirus vectors carrying the human JAM‐A. Their CM improved wound angiogenesis under hyperglycemia and enhanced diabetic wound repair, partially by increasing PDGF‐BB and VEGF expression.^118^

Nanoparticle loading into exosomes can lead to an increase in their targeting effect. For instance, superparamagnetic iron oxide nanoparticle‐labelled exosomes are intravenously injected into the circulatory system. The nanoparticles can be magnetised by using an external magnetic guide above the wound and act as a magnet‐guided navigation tool. They allow for better magnetic targeting to repair a wound, thus improving recovery efficiency.^119^ Optogenetic engineering of UCMSC‐exo is also possible. NO impacts collagen remodelling and repairs mechanical strength in wounds and one of the enzymes responsible for synthesis is NO synthase. The endothelial NO synthase promotes diabetic wound healing, which can be spontaneously loaded into UCMSC‐exo by EXPLOR, a blue light‐mediated reversible protein–protein interaction approach. Finally, this kind of endothelial NO synthase‐loaded exosome has been shown to remodel the immune microenvironment, which is beneficial for tissue repair.^120^

### 4.2 Administration and combined therapy of UCMSC secretome

In animal experiments, the medicine is typically administered subcutaneously around the wound bed for convenience and obviousness, which is called local injection. The medicine's solvent is usually NS or PBS. In mice and rats, 3–6 injection locations are generally chosen around the wound area, with 4 injection points being the most popular choice, that is, four sites on the border of the wound area are chosen to inject equal volumes of the reagent in the directions of east, west, north, and south.^121^ The effect of successive small doses is better than that of a single large dose. Several studies used intravenous injection or intradermal injection, but local injection has more benefits since it prevents immune cell phagocytosis, increases the bioavailability of the target tissue, and reduces the therapeutic dose.^13^ The average treatment period of these trials is focused on about 14 days.^49^, ^79^, ^93^

In clinical applications, the UCMSC secretome is employed in diverse ways, either in combination with biological or artificial scaffolds, or simply dissolved in NS or PBS. Scaffolds, like the secretome‐containing gel, are typically applied or injected into the wound, followed by protective fixation for the wound with a wound dressing or patch to maintain a moist and sterile environment.^58^, ^75^, ^76^ When directly dissolved, it can be infiltrated percutaneously or injected intradermally, such as using microneedles.^122^, ^123^

Common materials used for biological or artificial scaffolds are hydrogels, chitosan, hyaluronic acid (HA), and so forth. Application combined with scaffolds has more benefits than using them directly. Scaffolds can mimic the skin ECM to promote the growth and differentiation of cells and tissues. Moreover, scaffolds can provide a microenvironment suitable for cell adhesion, proliferation, and differentiation.^124^ The most widely accepted scaffolds are hydrogels, which promote proliferation via increasing cell growth rate, bone formation, and vascular anastomosis.^75^ There are various types of hydrogels, such as nano‐hydrogel, PF‐127 hydrogel, gelatin methacryloyl hydrogel, and silk fibroin and sericin composite hydrogel. All of the above hydrogels have been studied and employed as bioactive scaffolds for UCMSC‐exo to apply on the wound. Thus, they enhance curative efficiency and enrich the therapeutic roles of exosomes.^58^, ^125^, ^126^, ^127^ The scaffold can be chosen from HA gel. The loose, porous, biodegradable, and expandable microstructure of the HA gel offered a conducive microenvironment. Non‐invasive external treatment of types I and II diabetic foot ulcers can be achieved using UCMSC‐CM in HA gel.^128^ Methacrylated HA patches are a good alternative as well. These patches were produced through 3D bioprinting technology. In comparison to conventional scaffolds and carriers, methacrylated HA patches loaded with UCMSC‐exo exhibit better physical properties, swelling rates, degradation times, and biocompatibility in treatment, preserving the integrity and bioactivity of UCMSC‐exo.^129^

The secretome therapy combined with other medications and curative treatments can result in a greater efficacy than secretome treatment alone, and make a synergistic effect between drugs on wound healing promotion. For instance, the combination of UCMSC‐exo with aloe‐emodin can inhibit leishmaniasis parasites in addition to promoting wound healing.^130^ The combination of UCMSC‐exo and a novel microneedle, sponge spicules, could improve the efficiency of exosome skin absorption by forming microchannels and achieve more efficacy in photoaging reduction.^131^ In addition to combining microneedles, UCMSC‐exo can be combined with fractional laser and radiofrequency, and they have significantly improved the treatment of melasma (Table 1).^122^

### 4.3 Clinical therapeutic efficiency and biosafety of UCMSC secretome

The clinical application of the UCMSC secretome in skin rejuvenation is currently far more mature than in skin regeneration. In the field of skin regeneration, the UCMSC secretome is frequently employed in the clinical treatment of chronic wounds. One phase 2 clinical trial has demonstrated its effectiveness. A total of 41 chronic ulcers (diabetic and trophic ulcer) were administered topically by 10% UCMSC secretome gel, which lasted for 2 weeks and 1 month. It turns out that the considerable reduction in the length, width, and area of the wound between the time of the UCMSC secretome gel intervention and the present. Moreover, there was no indication of either local or systemic adverse effects.^132^

In the field of skin rejuvenation, the secretome can treat alopecia. One study demonstrated that after 12 injections with UCMSC‐CM over 3 months, 86.6% of patients experienced a good improvement in alopecia and 92% were very satisfied with the treatment.^100^ In a case report from Korea treated with UCMSCs injections, three patients, two with alopecia areata and one with alopecia universalis, were treated. After they received 2–15 rounds of injection treatment over 1–12 months, basically hair growth started after the first round, hair loss was alleviated at 3 months, and hair loss did not reoccur after the cure.^123^

Moreover, the secretome can brighten the skin, enhance the skin texture, and remove melasma. The general therapy approach needs to be combined with microneedling. In one report, 30 patients with an average age of 41 were selected to receive five treatments at 2‐week intervals. As a result, there was a significant decrease in the number of melanin deposits on the face, a reduction in wrinkles and pores, and an increase in skin elasticity.^105^ Another study showed effective alleviation of melasma in patients after 4 treatments each 1 month apart. They also compared the results of the secretome treatment combined with microneedling, fractional laser, and radiofrequency, with the highest patient satisfaction of 86.7% combined with radiofrequency.^122^ None of the aforementioned studies reveal any adverse effects brought on by the secretome.

The UCMSC secretome also has many defects:

1. There are some uncertainties in its function and composition. On the one hand, its effects are influenced by the secretory components and content of its donor cells. On the other hand, the secretome can exert different effects at different stages and has a double‐sided influence. For instance, if TGF‐β1 is overexpressed in UCMSC‐CM due to individual differences in the donor or variations in culture and extraction methods, it can affect scar formation during the later stages of wound healing, but normal amounts can promote angiogenesis.^35^, ^84^ Exosomal protein 14‐3‐3ζ not only acts as an ‘accelerator’ to repair skin during the proliferation stage but also works as a ‘brake’ during the remodelling stage.^61^ Furthermore, the main determinant of UCMSCs' ability to promote cutaneous wound healing is the donor's age.^13^
2. Long‐term anti‐inflammatory treatment of the wound is prone to foster infection. Given the anti‐inflammatory action of the UCMSC secretome, further attention is required to keep wounds sterile, especially chronic wounds.
3. Although the negative consequences of the UCMSC secretome are rarely discussed in the research, the issue of its biosafety for drug development still requires attention. Some reports mention the risk that the secretome may contain carcinogenic components. Because the secretome can suppress inflammatory cells and enhance angiogenesis, it is more likely to reduce the human body's immunocompetence against tumour cells and promote tumour angiogenesis.^13^, ^18^

## 5 SUMMARY AND OUTLOOK

The purpose of this review is to discuss the mechanism of UCMSC secretome to promote skin regeneration and rejuvenation, as well as the summary of related experimental methodologies and the prospect of clinical applications.

We can find a wide range of applications for the UCMSC secretome. It can be used as a therapeutic drug by itself, as a vehicle for active ingredients, or in combination with other treatments to promote skin regeneration. For example, it can be applied as a carrier for antimicrobial peptides, which can complement its own ability to play an antimicrobial role. Or, it can be used through modification or specialised culture, such as 3D culture or hypoxic condition culture, which will strengthen its ability to combat oxidative stress and gain other properties. It is also possible to construct UCMSC‐exo‐loaded carriers or change drug delivery, such as hydrogel, to make a colloidal solvent for the drug. And fix the medication in the form of a wound dressing or patch at the wound area, additionally switching from the subcutaneous injection delivery method to topical skin delivery. These increase the durability and stability of the secretome. Furthermore, it is more practical clinically, lowering the risk of infection during invasive procedures and providing protective fixation of the wound. In clinical practice, it tends to be used in medical settings for long‐term treatments such as the management of chronic wounds, hair loss, and medical beauty.

The effects of the UCMSC secretome on skin regeneration and rejuvenation are mainly divided into three aspects: wound healing, anti‐aging, and hair growth. Among these, the promotion of wound healing is the most important. Its effects and mechanisms on skin regeneration and rejuvenation can be totally summarised in the following six aspects:

1. The secretome can induce the initiation of wound healing and migration of keratinocytes during the haemostasis stage.
2. The secretome mainly plays an inhibitory role during the inflammation stage: it downregulates STAT3, TLR4, and NF‐κB signalling pathways, attenuates the activation and proliferation of leukocytes, T cells, and dendritic cells, and enhances the conversion of macrophages from M1 to M2. Thus, it reduces the secretion of inflammatory factors such as IL‐1β, IL‐2, IL‐6, IL‐17, IL‐23, TNF‐α, and IFN‐γ, and increases the release of anti‐inflammatory factors such as IL‐10, totally exerting an inhibitory effect on inflammation.
3. The secretome has the most critical role in the proliferation stage, mainly acting as a booster: it upregulates VEGF, ERK, MAPK, Ang/Tie, Wnt/β‐catenin, TGF‐β/Smad signalling pathways, increases the migration and proliferation of vascular endothelial cells, fibroblasts, and keratinocytes, and accelerates the transformation of fibroblasts into myofibroblasts, thereby enhancing collagen synthesis, ECM deposition, angiogenesis, nerve regeneration, and re‐epithelialization, thus serving as a pro‐proliferative and repair function for wounds.
4. The secretome reduces fibroblast‐to‐myofibroblast transition by inhibiting TGF‐β/Smad and Wnt/β‐catenin signalling pathways during the remodelling stage, which decreases collagen synthesis, ECM deposition, and prognostic scar formation.
5. The secretome in chronic wounds not only has the above similar effects, but also induces macrophage polarisation, regulates the ability of the skin cells to cope with oxidative stress damage, and reduces hypoxia‐induced apoptosis, thus enhancing angiogenesis and improving local microcirculation.
6. The secretome has plentiful roles in skin rejuvenation. The anti‐aging aspect acts to resist UV‐induced DNA damage and apoptosis in skin cells and produces antioxidant and anti‐inflammatory effects in the process. The secretome also whitens, anti‐wrinkle, reduces skin sensitivity, and protects the skin barrier. At last, the secretome promotes hair follicle formation to treat alopecia.

There are still many controversial aspects of UCMSC secretome on skin regeneration and rejuvenation. For instance, it is still unclear how the secretome modulates the VEGF signalling pathway to promote angiogenesis. The complex composition of the secretome is susceptible to donor effects leading to concerns about its biosafety.

In the final analysis, it has been demonstrated that the UCMSC secretome has outstanding capabilities for skin regeneration and rejuvenation, particularly for promoting wound healing. These abilities are made possible by the promotion of cell proliferation and angiogenesis, the development of hair follicles, and its anti‐inflammatory, anti‐fibrotic, anti‐oxidative, and anti‐aging properties. We believe that in the not‐too‐distant future, the UCMSC secretome can be well applied as a novel medication to meet the needs of skin regeneration and rejuvenation for a wide spectrum of patients in the clinic.

## AUTHOR CONTRIBUTIONS

Xixian Li drafted the paper and schematics. Dan Zhang and Yang Yu helped to prepare for this work. Liang Wang and Muxin Zhao revised and approved the final version.

## FUNDING INFORMATION

This work was supported by the Dalian Medical University‐Dalian Institute of Chemical Physics United Innovation Fund (NO. DMU‐2&DICP UN202311).

## CONFLICT OF INTEREST STATEMENT

The authors declare no conflicts of interest.

## Data Availability

Data sharing is not applicable to this article as no new data were created or analyzed in this study.
